# Fast and non-invasive PCR sexing of primates: apes, Old World monkeys, New World monkeys and Strepsirrhines

**DOI:** 10.1186/1472-6785-6-8

**Published:** 2006-06-08

**Authors:** Palle Villesen, Tina Fredsted

**Affiliations:** 1 Bioinformatics Research Center (BiRC), University of Aarhus, H. Guldbergsgade 10, building 1090, DK-8000 Aarhus C, Denmark; 2 Department of Ecology and Genetics, Institute of Biological Sciences, University of Aarhus, Ny Munkegade, building 1540, DK-8000 Aarhus C, Denmark

## Abstract

**Background:**

One of the key tools for determining the social structure of wild and endangered primates is the ability to sex DNA from small amounts of non-invasive samples that are likely to include highly degraded DNA. Traditional markers for molecular sex determination of primates are developed on the basis of the human sequence and are often non-functional in distantly related primate species. Hence, it is highly desirable to develop markers that simultaneously detect Y- and X-chromosome specific sequences and also work across many species.

**Results:**

A novel method for sex identification in primates is described using a triple primer PCR reaction and agarose gel electrophoresis of the sex-chromosomal isoforms of the ubiquitously transcribed tetratricopeptide repeat protein gene (*UTX*/*UTY*). By comparing genomic data from several mammals we identified the *UTX*/*UTY *locus as the best candidate for a universal primate sexing marker. Using data from several species we identified a XY-conserved region, a Y conserved region and an X conserved region. This enabled the design of a triple primer PCR setup that amplifies X and Y products of different length in a single PCR reaction.

**Conclusion:**

This simple PCR amplification of X and Y fragments is useful for sexing DNA samples from all species of primates. Furthermore, since the amplified fragments are very short the method can be applied to fragmented DNA extracted from non-invasive samples.

## Background

The ability to identify the sex of a DNA sample is an important tool in molecular ecology and conservation genetics. The optimal marker would work on small amounts of non-invasive samples that are likely to include highly degraded DNA and be applicable in many species.

Molecular sex identification normally works by PCR amplification of sex specific regions that differ in length. The PCR products can then be visualized using standard electrophoresis, revealing females as homozygotes (XX) and males as heterozygotes (XY). Generally, in order to detect the male sex, a Y chromosomal fragment must be amplified. This can be done by (a) amplification of a homolog region on X and Y with a length difference, (b) a triple primer PCR with a common primer X/Y and a Y specific primer (short fragment) and X specific primer (longer fragment) or (c) a multiplex PCR with a Y region and an positive control (autosomal or X region).

Several loci have been used for sex identification in humans and closely related species, e.g. the amelogenin system [1-3] the zinc-finger protein [4,5], the SRY locus [6] or a combination [7]. Detection of sex-specific restriction patterns [8] requires some pre-analysis development (i.e. sequencing to identify restriction sites) and enzyme restriction of PCR products, which is time consuming. The SRY locus requires co-amplification of external control regions [6,7], which may be unreliable for non-invasive DNA samples with DNA of low quality.

The widely used amelogenin system [1] has recently been found to provide ambiguous results in humans such as null-alleles, primer mutations or amplification failure, resulting in erroneous gender determination [9-11]. It works in closely related apes [2], but not orang-utans [6], baboons or more distantly related species [12]. Also, the Sullivan amelogenin system amplifies a very small X-Y size difference (6 bp), which does not consistently resolve well on agarose gels. Therefore this system needs more time-consuming and expensive acrylamide gel- or capillary electrophoresis. Recently, a new general multiplexing method was published, using the amelogenin locus, the SRY locus and group specific primers and suitable for non-invasive samples [7]. Non-identification of males may result from non-amplification of the Y fragment and it has been argued [13] that all "females" (XX or XY with no Y amplification) should be verified with a second independent sex test. This calls for the development of multiple independent tests that can be carried out in parallel.

We have previously developed a new primer pair for a different region of the amelogenin gene suitable for sexing lemurs and humans and therefore possibly most primate species [3]. However, like the zing-finger protein system [4], the resulting fragments are too long (> 250 bp) for non-invasive samples, which often contain highly degraded DNA. Alternatively, we then designed primers for a small region of the DEAD-BOX gene, which are able to sex apes and monkeys, but these primers do not work in prosimians – probably due to primer region mutations [14].

At present, genomic sequence information is only available for a few primates (human, chimpanzee, macaque) and rodents (mouse, rat) or even more distantly related species. So far, we find that it is impossible to identify suitable homolog XY regions with the desired degree of conservation for a standard two-primer PCR design that will work in all primates.

The objective of this study was to identify a suitable region for a triple primer PCR design with a shared XY primer in combination with an X- and Y-specific primer. The primers should amplify a short region and be widely conserved through primate evolution, such that the method would work on non-invasive samples from all primate species.

## Results

### A region of *UTY *is highly conserved between human and mouse

The comparison of the repeat masked human, chimpanzee and mouse Y chromosome yielded surprisingly few results. The best hit was 248 bp with 95.56% identity between mouse and human. This region is a part of the Y-chromosomal isoform of the ubiquitously transcribed tetratricopeptide repeat protein gene (*UTY*). Hence, *UTY *and the X-chromosomal homolog (*UTX*) were selected for further analysis.

### The UTX/UTY region is suitable for triple primer design

**Figure 1:**
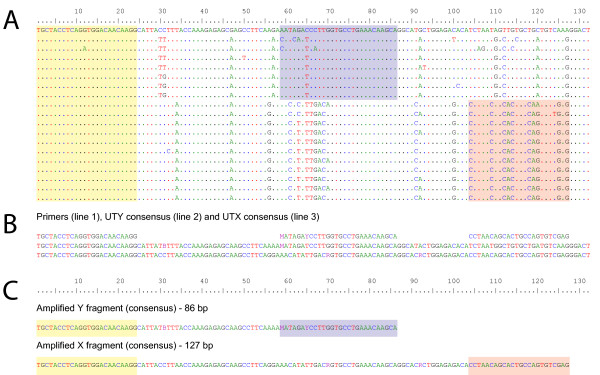
**UTY/UTX alignment**.A) Alignment of primate *UTX*/*UTY *regions. B) Top: Primers. Middle: *UTY *consensus sequence. Bottom: *UTX *consensus sequence. C) The resulting consensus fragments amplified by the three primers.

A small region in the alignment of 19 primate *UTX*/*UTY *sequences (see methods) showed three distinct patterns, useful for primer design (figure 1): The *UTX*/*UTY *primer is located in a region with high conservation between primate X and Y homologs (marked in green in figure 1A). The *UTY *primer is located in a region conserved between Y homologs but with conserved differences between the X and Y homologs (marked in blue in figure 1A) and the *UTX *primer is located in a region conserved between X homologs but with conserved differences between the X and Y homologs (marked in red in figure 1A). The resulting primers (Table 1, figure 1B) were chosen to be able to bind to all sequences in the alignment and to yield both short fragment sizes as well as fragments with differing lengths. The two fragments (consensus fragment length: Y = 86 bp, X = 127 bp) are easily separated on agarose gels (figure 1C).

### Successful sexing of all primate species tested

**Figure 2:**
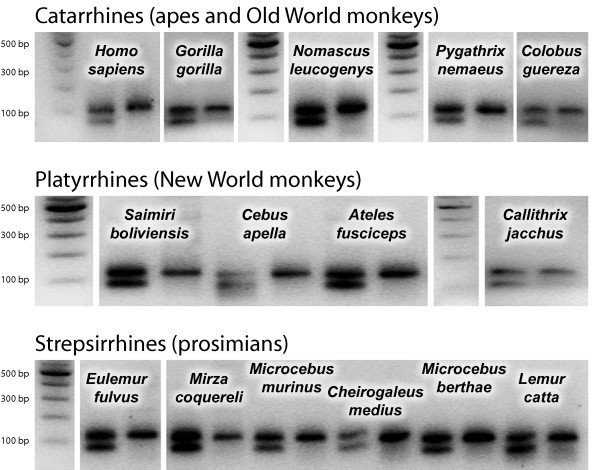
**Successful sexing in XX primate species**. The primers were successfully tested in apes, New World monkeys, Old World monkeys and prosimians using identical PCR conditions. Males: two bands (XY), females: one band (XX).

The primers work in all species tested yielding amplifications of both X and Y fragments (figure 2). Amplifications were successful regardless of template DNA concentration, hence the method is suitable for analysing non-invasive samples.

Since the primers were designed in well conserved regions, no species-specific optimization was necessary and a shared PCR protocol and identical annealing temperature was used (table 1).

## Discussion

In this study we have established a simple, accurate and widely applicable method for determining the sex of primate DNA samples by using triple primer PCR of a small region of the *UTX*/*UTY *gene. The ubiquitously transcribed tetratricopeptide repeat protein gene (*UTX*/*UTY*) is located on the X and Y chromosomes and our analysis identified a region in the human *UTY *having the highest identity to the mouse Y chromosome. Due to the high conservation of this region the triple primer PCR setup works in all primate species tested. Furthermore, the method contains an internal positive control (the shared primer), but should always be tested in samples of known sex before actual analysis is carried out. Also, it may be necessary to perform species specific optimization of annealing temperature and/or primer concentrations prior to analysis.

Since the nature of the Y chromosome allows deletion of many regions, it can be expected that with an increased number of individuals tested deletion of UTY might be found in low frequency (as reported for amelogenin-Y [15]). Furthermore, primer region mutations may result in non-identification of males due to PCR failure, which are known in the amelogenin system [9-11]. Generally, non-identification of males may result from non-amplification of the Y fragment and it has been argued [13] that all "females" (XX or XY with no Y amplification) should be analysed with at least two independent sex tests. It is now possible to combine the analysis of amelogenin, SRY and UTX/UTY in parallel for most primate species, thereby obtaining reliable sex determination (e.g. [7] and this study) using independent PCR runs. Given the low amount of DNA usually obtained from non-invasive sampling, the optimal approach for molecular sexing would be to run a single multiplex PCR analysis, but such a protocol has not been developed yet.

## Conclusion

Our findings show that the PCR assay based on the *UTX*/*UTY *gene is reliable for sex identification in primates. The advantage of this assay is the extreme primer conservation and wide applicability and ease of use. In combination with other single locus markers, it is now possible to reliably sex individuals of most (if not all) primate species.

## Methods

### Primer region identification and design

Primate Y chromosomes were downloaded from Ensembl [16] and compared to the mouse Y chromosome using BLAST with strict parameters (W = 18 (primer length), e = 1E-100). The region with the highest identity was found to be in the *UTY *gene in humans (248 bp with 95.56% identity to mouse, Human Y: 13909061-13909308), and this gene and the X homolog (*UTX*) was selected for further analysis.

8 *UTY *and 11 *UTX *sequences were downloaded from GenBank and aligned (*UTY*: *Eulemur fulvus, Macaca fascicularis, Ateles geoffroyi, Hylobates lar, Pongo pygmaeus, Gorilla gorilla, Pan troglodytes, Homo sapiens UTX*: *Lemur catta, Cheirogaleus medius, Colobus sp., Hylobates lar, Presbytis entellus, Leontopithecus rosalia, Ateles geoffroyi, Macaca fascicularis, Gorilla gorilla, Pongo pygmaeus, Homo sapiens*. The alignment files are available as additional files 1, 2).

Adequate regions were selected manually (figure 1A) and degenerate primers were designed using the Primo online software [17]. The resulting primers were designed with relatively high annealing temperatures to allow possible mismatches to the template in distantly related species (table 1).

### DNA extraction and PCR

DNA was extracted from shed or plucked hairs, ear tissue or blood. From hair samples DNA was extracted using a slight modification of the Chelex protocol [18]: Approximately 5 mm of the root portion of 5–8 hairs from each individual was cut directly into 1.5 mL Eppendorf tubes containing 200 μl of 20% Chelex resin solution (BIO-Rad, Hercules, CA, USA). Tubes were vortexed briefly, boiled for 12 min, centrifuged 3 min at 13000 rpm and stored at minus 20°C. From tissue samples DNA was extracted using DNeasy Tissue Kit (cat. no. 69506, Qiagen), and from blood samples DNA was extracted using proteinase K digestion, followed by a protein salting-out step as described in [19]. PCR was performed in a total volume of 10 μl and contained 1 μl buffer (1.5 mM MgCl_2_), 1.6 μl dNTP (1.25 mM of A, C, G, T, respectively), 4, 1 and 0.25 μl of UTY, UTXY and UTX primers (10 pmol/μl), 0.1 μl *Taq *polymerase (Amersham Pharmacia Biotech), topped up with distilled water to 8 μl and 2 μl template DNA was added. The different volumes of the three primers were found after optimization for equal intensity of the X and Y fragments. Cycling conditions were 94°C for 3 min, and 35 cycles of 94°C for 30 s, 57°C for 40 s and 72°C for 1 min and a final extension step of 72°C for 7 min. PCR fragments were separated on 2 % agarose gels (120 V, 1 hour 45 minutes) and visualized using SybrGreen staining.

## Authors' contributions

PV carried out the sequence analysis, designed the primers and drafted the manuscript. TF obtained primate samples from international contacts, extracted DNA, carried out all the lab-work and drafted the manuscript.

## Supplementary Material

Additional File 1 Full *UTX*/*UTY *sequence set. The unaligned set (fasta format) of 8 *UTY *and 11 *UTX *sequences (*UTY*: *Eulemur fulvus, Macaca fascicularis, Ateles geoffroyi, Hylobates lar, Pongo pygmaeus, Gorilla gorilla, Pan troglodytes, Homo sapiens UTX*: *Lemur catta, Cheirogaleus medius, Colobus sp., Hylobates lar, Presbytis entellus, Leontopithecus rosalia, Ateles geoffroyi, Macaca fascicularis, Gorilla gorilla, Pongo pygmaeus, Homo sapiens*). Click here for file

Additional File 2 Cropped *UTX*/*UTY *alignment. The cropped alignment (fasta format) of 8 UTY and 11 *UTX *sequences (see also figure 1A, *UTY*: *Eulemur fulvus, Macaca fascicularis, Ateles geoffroyi, Hylobates lar, Pongo pygmaeus, Gorilla gorilla, Pan troglodytes, Homo sapiens UTX*: *Lemur catta, Cheirogaleus medius, Colobus sp., Hylobates lar, Presbytis entellus, Leontopithecus rosalia, Ateles geoffroyi, Macaca fascicularis, Gorilla gorilla, Pongo pygmaeus, Homo sapiens*). Click here for file

## Figures and Tables

**Table 1:** PCR primers, degeneracy and annealing temperatures (Ta).

Primer name	Primer sequence (5'-3')^1^	Degeneracy	T_a_°C
UTX/UTY	TGCTACCTCAGGTGGACAACAAGG	1	62
UTY	TGCTTGTTTCAGGCACCAAGG**R**TCTAT**K**	4	62
UTX	CTCGACACTGGCAGTGCTGTTAGG	1	62

^1^Degenerate nucleotides are shown in bold.
